# 145. Harnessing the Inoculum Effect to Diagnose Molecular Mechanisms of Carbapenem Resistance

**DOI:** 10.1093/ofid/ofac492.223

**Published:** 2022-12-15

**Authors:** Alexis Jaramillo Cartagena, Kyra Taylor, Roby P Bhattacharyya

**Affiliations:** Broad Institute of MIT and Harvard, Cambridge, Massachusetts; Broad Institute of MIT and Harvard, Cambridge, Massachusetts; Massachusetts General Hospital, Cambridge, Massachusetts

## Abstract

**Background:**

The global spread of carbapenem-resistant *Enterobacterales* (CRE) presents a major threat to public health. CRE employ two molecular mechanisms to evade carbapenems: 1) expression carbapenemases (CPases), which efficiently hydrolyze carbapenems or 2) disruption of porins, which reduces carbapenem influx to levels that can be hydrolyzed by certain beta-lactamases. Diagnosing mechanisms underlying carbapenem resistance is important for infection control and to administer appropriate treatments.

**Methods:**

We measured Meropenem and Ertapenem minimum inhibitory concentrations (MICs) for 103 clinical *Enterobacterales* isolated from hospitals in Massachusetts and California using broth microdilution assays at 14 inocula spanning four orders of magnitude. They represented 30 isolates encoding CPases and intact porins, 46 isolates with disruptions in one or both of the porins responsible for carbapenem influx (OmpC and OmpF), 25 encoding CPases with disrupted porins, and two controls encoding intact porins and no CPases.

**Results:**

We observed that the two mechanisms result in distinct profiles; first the carbapenem MICs of CPase-encoding strains show a strong inoculum dependence (Fig 1.A), whereas the MICs of porin deficient strains remain largely constant at all inocula (Fig 1.B). The synergistic action of these two mechanisms leads to high-level resistance that we termed “hyper-CRE” (Fig 1.C). Together these factors explain the level of resistance in nearly all our CRE isolates. To validate the hyper-CRE phenotype, we successfully employed CRISPR-based gene editing to show that knocking out the major porin in CPase-producing strains elevates their carbapenem resistance to hyper-CRE levels.

We also determined 18% of our isolates changed susceptibility classification within the Clinical Laboratory and Standards Institute (CLSI) recommended inoculum range. This is worrisome for the treatment of infections with strains that are deemed susceptible via *in vitro* AST assays but are truly resistant *in vivo*.

**Figure:**
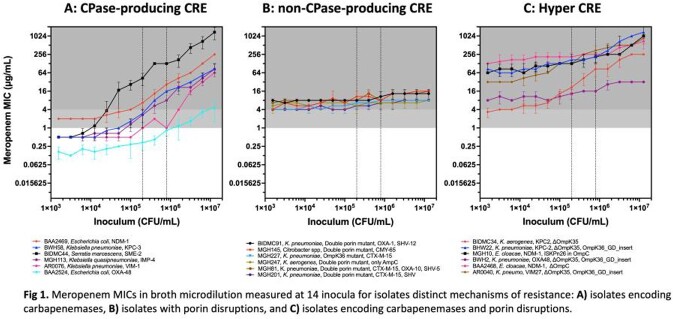

**Conclusion:**

Overall, our approach has demonstrated that measuring MICs at different inoculum can yield crucial diagnostic information about mechanisms of resistance which has important implications for patient care, infection control, and surveillance of emerging CPases.

**Disclosures:**

**All Authors**: No reported disclosures.

